# Advanced 3D “Modeling” and “Printing” for the Surgical Planning of a Successful Case of Thoraco-Omphalopagus Conjoined Twins Separation

**DOI:** 10.3389/fphys.2020.566766

**Published:** 2020-11-13

**Authors:** Alessandro Inserra, Luca Borro, Marco Spada, Simone Frediani, Aurelio Secinaro

**Affiliations:** ^1^Department of General Surgery, Bambino Gesù Children’s Hospital, Rome, Italy; ^2^Department of Imaging, Bambino Gesù Children’s Hospital, Rome, Italy

**Keywords:** 3D printing, conjoined twins, 3D printing in cardiothoracic surgery, 3D images, 3D printing in surgery

## Abstract

**Background:**

The surgical separation of two Conjoined Twins is a particularly complex operation. Surgical times are particularly long and post-operative complications are very frequent in this type of procedure. We report a clinical case of surgical separation of two thoraco-omphalopagus conjoined twins in which, thanks to the use of (3D) three dimensional technologies, we were able to significantly reduce operative times and improve clinical outcomes.

**Methods:**

We performed a 3D reconstruction of the anatomical parts involved in congenital fusion using Computer Tomography (CT) images.

We obtained virtual anatomical models of the patients which allowed us to estimate essential details as the residual post-operative thoracic volume as well as the exact position of resection planes for both the general separation and for the hepatic splitting procedure. Subsequently, we printed 3D anatomical models of the thoracic cage and sternum and of the liver with the plane of resection. Finally, we printed an additional 3D anatomical model of the two patients representing different organs with multiple colors and materials.

**Results:**

The use of 3D printing reduced the duration of surgery by 30% with a favorable patient outcome. Two years after the operation, the patients do not present any type of deficit and have a normal life without any significant complication.

**Conclusion:**

Virtual anatomical 3D models and 3D printing represent a valid technological tool to support complex surgical operations, especially in pre-surgical planning. 3D models are important tools to better understand complex anatomy and to discuss clinical cases among members of the surgical team.

## Highlights

- A multidisciplinary team in can increase the chances of success in cases of complex surgery;
- The use of new technologies such as 3D printing can lead to a substantial reduction of the operating times and of the risks for patients;
- Pre-operative planning helped by technologically advanced 3D tools gives an important contribution to the technical preparation of the surgical team.

## Introduction

Virtual pre-operative planning tools such as Virtual Reality or rapid prototyping of physical objects (Guerriero et al., 2018) were successfully applied to several medical fields. Interestingly, many studies evaluated the positive impact of Virtual Reality in different surgical disciplines both for practicing and training/education purposes (Jensen et al., 2015). Three dimensional (3D) printing also received significant consideration, especially as a pre-operative planning tool (Mitsouras et al., 2015).

Three dimensional technology applied to pre-operative planning is particularly useful in the study of complex clinical cases, which are more difficult to assess using only standard radiological methods.

One of the main advantages that 3D technology brings to pre-operative planning is a better understanding of the anatomy in particularly complex cases.

Based on these considerations, we decided to apply 3D technology to support all the clinical/surgical steps needed to plan and complete the separation of thoraco-omphalopagus conjoined twins.

Healthy 6-month-old thoraco-omphalopagus twins were referred to our institution from abroad. Pre-operative investigations included chest and abdominal X-Rays and contrast-enhanced head-to-pelvis computed tomography (CT) (Trost et al., 2016). CT was performed under general anesthesia showing two separate cardiovascular systems with normally structured hearts, a common pericardial sac, “arch-shaped” sternums, anteriorly fused diaphragms, supraumbilical peritoneal communication with disconnected digestive systems, and two normally structured livers largely fused anteriorly, with no evidence of arterial, portal, and biliary cross-circulation.

**FIGURE 1:**
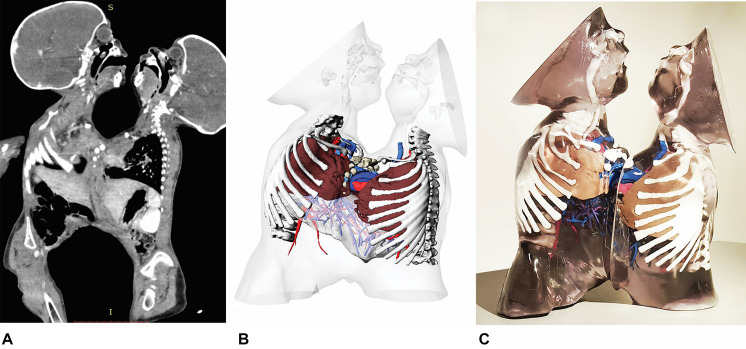
CT sagittal images of the entire conjoined anatomical structures **(A)**, 3D virtual reconstruction of patient’s anatomy **(B)**, multi-color and multi-material 3D printed anatomical model **(C)**.

In order to provide additional anatomical information to conventional radiological images, 3D technology was used to complement CT data (Shen et al., 2017). Two dimensional (2D) radiological images and 3D virtual rendering (VR) reconstructions are normally employed before surgery in complex surgical instances. However, in this case it was often difficult to interpret spatial anatomical details and relationships between congenital defects only using conventional imaging tools (Figure 1A). Therefore, advanced 3D reconstructions were applied to better represent the anatomical structures involved in the congenital fusion (Figure 1B). Additionally, they were employed to simulate part of the surgical process (e.g., rib cage closure) and to evaluate residual thoracic and hepatic volumes. 3D printed models provided tangible anatomical structures with different colors and materials. Both 3D virtual and printed models were shown and manipulated by the multidisciplinary surgical team during clinical meetings in order to analyze all relevant anatomical details and to display different operative scenarios, thus improving the clinical decision-making process. 3D prints were also used to communicate with the parents of the patients so as to illustrate the operational strategy chosen by the surgical staff, promoting family involvement and understanding as well as increasing the level of doctor-patient communication. This was especially relevant while obtaining informed consent before the operation.

## Methods

**FIGURE 2:**
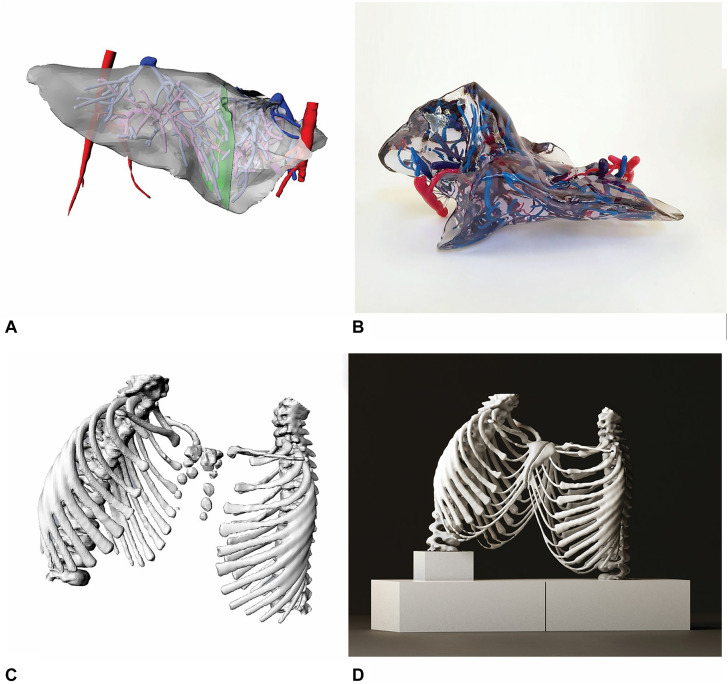
3D virtual reconstruction of liver and hepatic vascularization from CT images **(A)**, multi-color and multi-material 3D printed liver **(B)**. 3D model of the ribcage with conjoined sternum **(C)**, 3D print of the ribcage with white Polylactic Acid material **(D)**.

Segmentation was performed using a commercially available software (Mimics v.21, Materialise^®^, Belgium) and its tools:

• Focused 3D segmentation of the liver parenchyma including arterial and venous vascularization was performed to obtain a virtual transparent model and define the possible plane for hepatic splitting (Video). We usually employ co-registered CT and MRI images in this setting (Panigrahy et al., 2000; Tang et al., 2006), but in this particular case all the necessary clinical information was provided by CT only. The liver and hepatic vessels were reconstructed with a segmentation protocol that allowed the selection and superimposition of multiple phases (e.g., arterial, venous), regions of interests, vessel skeletonization, vascular analysis and segmental subdivision (Selle et al., 2002). Mimics’ “Thresholding” and “Multiple Slice Editing” tools. Were used for this purpose.
• For simulation of hepatic surgery, an optimal resection plane was identified on the 3D printed model (Figure 2B). This plane, through the use of anatomical landmarks, was reproduced on the 3D virtual model for the evaluation of postoperative residual hepatic volumes (Figure 2A).

• The upper airways and lungs were recreated with an automatic 3D reconstruction protocol using Mimics’ Pulmonary Module.
• The rib cage was reconstructed with a semi-automatic segmentation approach using Mimics’ Thresholding and Multiple Slice Editing tools. The complete 3D virtual and printed models were also used to simulate the surgical closure of the rib cage. Hence, it was possible to estimate residual thoracic volumes with the aim of assessing the effective postoperative lung functional capacity.
• The cardio-circulatory apparatus was reconstructed with a semi-automatic segmentation protocol with using Mimics’ “CT-Heart” module, based on a region-growing algorithm. The pericardium subject to congenital fusion was manually segmented based on CT images.

Virtual 3D models were post-processed with additional software (“3-Matic” v.21, Materialise^®^, Belgium; and “MeshMixer” v. 3.5, Autodesk^®^, United States) and converted to stereolithographic (STL) format to be sent to 3D printers (Supplementary Table 1).

With 3-Matic software we have carried out all the Boolean subtraction operations between the various anatomical parts in order to make the model compatible for 3D printing and free of topological mesh errors. In MeshMixer the “Inspector” tool was used to detect and close all open surfaces of the model. The “Plane-Cut” tool was employed to make virtual cuts and resect unnecessary 3D anatomical segments.

Three 3D models were created, of the liver, rib cage (Figure 2C), and whole thoraco-abdominal region, respectively. All subparts were separated by one another and marked with a different color. This allowed to obtain a 3D print providing an easy understanding of the final result (Figure 1C). To perform this complex separation with non-self-intersecting mesh surfaces, repeated boolean subtraction operations were carried out. Then, all the subparts were saved in different STL files, each with the same spatial coordinates and same origin of the axes, and were sent to the 3D printer. The liver model consisted of the hepatic parenchyma, hepatic vascularization and bile ducts. The parenchyma was printed using a transparent rigid resin, while the hepatic vascularization was produced with the use resins of three different color (red for the arteries, pink for the portal system, and blue for the hepatic veins). The 3D model of the rib cage was reconstructed and printed with double extrusion Fusion Filament Fabrication (FFF) technology (Figure 2D), using white thermoplastic filament of Polylactic Acid (PLA) and Polyvinyl Alcohol (PVA) water soluble support on a Fortus 380 mc 3D Printer (Stratasys^®^, United States) with 0.3 mm nozzle diameter. The model was used to define a resection plane.

A 100% concentric pattern infill was employed for this model with four perimeter layers and a layer thickness of 0.1 mm. The thoraco-abdominal model consisted of a volume delimited by the skin, in which all organs were contained. The whole thoraco-abdominal 3D morphology was printed by using transparent and multicolor resins on a Stratasys J750 3D Printer (Stratasys^®^, United States), obtaining two separate results according to the pre-defined resection planes.

All models were 3D printed as unique whole pieces without any post-production assembly operation. All 3D printed models had an unaltered polygonal mesh compared to what was obtained by Mimics.

Based on the complementary information of CT imaging and 3D printing, the twins underwent skin expander placement in the thoraco-abdominal wall. Surgical separation was performed 6 months later, with reconstruction of skin defects according to Sun et al. (2008), using a Porous High-Density Polyethylene (Medpore) plate with a surface of about 23,5 cm^2^ for each patient.

## Results

The surgical procedure was uncomplicated and the twins were discharged from the hospital in good clinical condition after healing.

The surgical time for the separation of the twins was 4 h and 57 min, about 3 h less than other similar cases reported in the literature (Saranrittichai et al., 2007; Bugaje et al., 2010). There were no intra and postoperative complications such as bleeding and need for transfusions unlike similar clinical cases described in the literature (Zhong et al., 2013), no blood transfusions were required. Post-surgery intubation times were 10 days and thoracic-abdominal drainages were removed after 5 days from surgery.

Three dimensional reconstructions of anatomical models were performed multiple times based on the specific needs expressed by the surgical team and on the availability of the radiological exams performed on the patients. 3D printing required repeated model optimization in order to perform seamless printing. 3D reconstructions of anatomical models took about 6 months, while 3D printing of the rib cage, liver and the entire thoraco-abdominal anatomy was completed in 12, 8, and 24 h, respectively. Unlike other similar cases reported in the literature (Shen et al., 2017), the 3D printing process of our anatomical models was carried out directly without the use of cast and molds. This reduced production times. The multi-color and multi-material 3D printing technology allowed us to print the skin surface with transparent material so that we could look inside the model and study the mutual position of abdominal organs, which were printed in different colors to make them recognizable.

In previous works, the skin surface was printed with opaque material and the internal organs were not color-coded (Wood et al., 2017). 3D reconstructions obtained from CT images allowed us to quantify the chest volumes of the twins, which were 43,162 cm^3^ and 29,420 cm^3^ in the pre-operative phase and 61,342 cm^3^ and 60,183 cm^3^ 1 month after surgery, respectively. The initial liver volume measured at preoperative CT was 63,440 cm^3^ while the two livers volumes measured 1 year after surgical separation were of 33,263 cm^3^ and 31,103 cm^3^, respectively. Both twins were recently seen at 3 years follow-up and are healing well from surgery. All the specialists involved evaluated the twins and found both in good clinical condition, with regular growth and psychomotor development.

Also the thorax CT and Abdominal United States were normal.

## Discussion

Surgical separation of conjoined twins requires the joint efforts of a multidisciplinary team comprising various specialists including plastic, abdominal and cardiothoracic surgeons.

3D virtual and printing models played a crucial role in our case, as the surgical team used these models for 1 year during pre-operative meetings to obtain important information on anatomical details and to discuss different interventional strategies. Both 3D printing and Virtual 3D Modeling had an important role in the understanding of complex anatomy and in the joint surgical discussion of the clinical case. The 3D printed rib cage model was very useful to all members of the surgical team for a better understanding of the CT radiological images. Above all, the 3D reconstructed bone model was able to highlight the geometry of the fused sternal arc, which is uncommon to see in routine clinical practice. This information was certainly visible in the conventional radiological images, but the reciprocal position of the patients’ rib cages was better represented with the use of 3D printed models.

The use of 3D printing technology is ideal for complex clinical cases such as the one we presented, because it increases the effectiveness of interdisciplinary communication, improves the assessment of complex anatomy and allows for a specific training of the surgical team directly on the pre-operative model. We recommend the use of 3D imagery and 3D printed models for use in complex clinical cases since it can increase the effectiveness of interdisciplinary clinical communication. To make 3D printing technology more suitable for clinical practice, however, it would be necessary to develop materials with tactile characteristics closer to human soft tissues.

3D-printed models with flexible materials and structures that can be assembled or taken apart into smaller substructures might be more useful in understanding of the thoracoabdominal anatomy.

Nevertheless, 3D printing of such complex anatomical models, composed of multiple anatomical parts joined together, makes it very difficult to use soft materials. For this reason, we were not been able to experiment with additional printing materials. Despite some limitations of the currently available printing system, 3D printing technology offers a tactile perception to the surgeon and this is the main difference between 3D printing and Virtual Reality. Surgeons greatly appreciated the ability to touch and feel the shape and orientation of the “physical” patient-specific anatomical model. Unlike the models consulted only via Virtual Reality technology, 3D printed models are easier to use during pre-operative meetings and during surgery. The models can be consulted by each specialist and discussed without the need to use computer tools.

The “standard” pre-operative process usually does not use 3D printed models. It involves the consultation of a series of radiological images such as CT, MRI and Ultrasound images. Sometimes, these images are post-processed to obtain 3D Volume Rendering reconstructions that allow for a non-immersive navigation of anatomical models compared to the visual and tactile experience provided by 3D printed models.

In complex clinical cases this is a strong limitation for a thorough understanding the spatial orientation of the anatomical structures and their relationship by the entire surgical team, which includes both medical and non-medical health workers.

Often, the available radiological images are discussed by the surgical team in pre-operative meetings where images are simply projected and viewed on a large screen.

We believe that the use of more advanced technologies, such as 3D printing or Virtual Reality, is a valuable clinical support to improve the standard pre-operative planning phase. Unlike the regular process, 3D-assisted planning conducted with patient-specific 3D printed models allows the whole team to better analyze the clinical case and may be useful to simulate surgery directly on the model. This is important for both the surgical team in the pre-operative phase and for young surgeons during their training.

In conclusion, the use of 3D printing technology as a means to complement the information provided by CT scans offers useful and reliable information for planning difficult surgical procedures such as the separation of conjoined twins.

Our experience using 3D printing in conjoined twins surgery is limited to a single clinical case, but we can confirm that the use of 3D printing in the pre-operative phase solves a number of clinical, technical and logistical problems. The 3D models used in this clinical case were extremely useful for the set-up of the operating room, allowing us to organize the spaces appropriately. Thanks to the placement of the 3D printed models on the operating bed just a few days before surgery, it was possible to check that all the organized spaces were adequate according to the spatial orientation of the patients on the operating bed.

The ability to practice surgical interventions using these anatomical models significantly reduced the operating time, reducing both costs, and patient risk.

## Data Availability Statement

The raw data supporting the conclusions of this article will be made available by the authors, without undue reservation.

## Ethics Statement

The studies involving human participants were reviewed and approved by the Bambino Gesù Ethics Committee. Written informed consent to participate in this study was provided by the participants’ legal guardian/next of kin. Written informed consent was obtained from the minor(s)’ legal guardian/next of kin for the publication of any potentially identifiable images or data included in this article.

## Author Contributions

AI and MS: writing the article and reviews of results. LB and SF: writing the article, images, and video production. AS: writing the article, reviews of results. All authours contributed to the article and approved the submitted version.

## Conflict of Interest

The authors declare that the research was conducted in the absence of any commercial or financial relationships that could be construed as a potential conflict of interest.

## Supplementary Material

Supplementary Table 1 Detailed description of the 3D segmentation and post-processing anatomical models with different software (Mimics v.21, 3-Matica v.21, and MeshMixer vers. 3.5).
